# Poly-dA:dT Tracts Form an *In Vivo* Nucleosomal Turnstile

**DOI:** 10.1371/journal.pone.0110479

**Published:** 2014-10-29

**Authors:** Carl G. de Boer, Timothy R. Hughes

**Affiliations:** 1 Department of Molecular Genetics, University of Toronto, Toronto, Ontario, Canada; 2 Donnelly Centre for Cellular and Biomolecular Research, University of Toronto, Toronto, Ontario, Canada; Texas A&M University, United States of America

## Abstract

Nucleosomes regulate many DNA-dependent processes by controlling the accessibility of DNA, and DNA sequences such as the poly-dA:dT element are known to affect nucleosome binding. We demonstrate that poly-dA:dT tracts form an asymmetric barrier to nucleosome movement *in vivo*, mediated by ATP-dependent chromatin remodelers. We theorize that nucleosome transit over poly-A elements is more energetically favourable in one direction, leading to an asymmetric arrangement of nucleosomes around these sequences. We demonstrate that different arrangements of poly-A and poly-T tracts result in very different outcomes for nucleosome occupancy in yeast, mouse, and human, and show that yeast takes advantage of this phenomenon in its promoter architecture.

## Introduction

*In vivo*, promoters are characterized by a nucleosome free region (NFR) that is followed by a periodic phasing of well-positioned nucleosomes continuing into the gene body. In yeast, this phasing is absent *in vitro*, but can be restored by the addition of a whole cell extract (WCE) and ATP, presumably a result of ATP-dependent chromatin remodelers (CRs) [1]. The promoter NFR, however, is largely preserved *in vitro* because yeast promoters contain sequences that are inherently refractory to nucleosome formation, such as low G/C content [2] and poly-dA:dT tracts [3].

**Figure 1:**
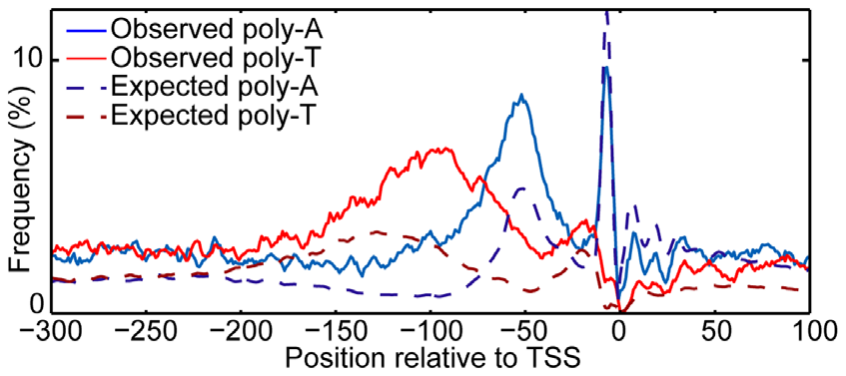
Yeast promoters have a biased distribution of poly-As and poly-Ts. The observed and expected frequency of poly-A and poly-T (AAAAA/TTTTT) elements across yeast promoters is shown, with expected calculated given the base content of the region. A greater number of poly-Ts and poly-As occur than expected in the −115:−75 and −75:−35 regions, respectively (p<10^−6^ by simulation; see methods).

Yeast promoters have a biased distribution of poly-A and poly-T elements flanking nucleosome free regions [4], [5], which cannot be explained solely by the biased base content (**Figure 1**). This asymmetric poly-A/poly-T arrangement has no known function and is incongruous with the model that poly-dA:dT tracts simply exclude nucleosomes via a rigid DNA structure [6] since the DNA should resist bending equally in either orientation.

## Results and Discussion

**Figure 2:**
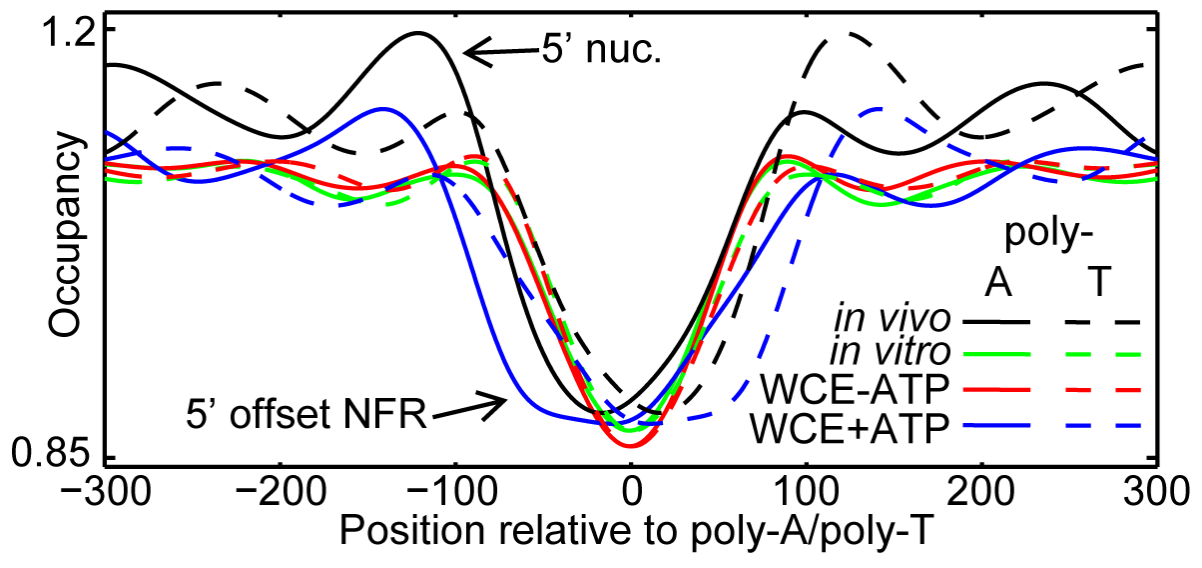
Nucleosomes are arranged asymmetrically around poly-dA:dT tracts. Average nucleosome occupancy surrounding poly-A and poly-T sequences (AAAAA/TTTTT) for salt gradient dialysis (*in vitro*), WCE without ATP (WCE-ATP), WCE with ATP added (WCE+ATP), as well as *in vivo* occupancy [1]. The difference in occupancy between poly-As and poly-Ts is significant only for *in vivo* and WCE+ATP (by rank sum; see **Figure S1** in **File S1**).

Hypothesizing that the asymmetric arrangement of these elements in promoters may have evolved to maintain promoter NFRs through some effect on nucleosome occupancy, we identified all non-overlapping poly-A sequences of exactly length five (AAAAA) in the yeast genome and analyzed the nucleosome occupancy [1] surrounding these elements (**Figure 2**). *In vitro*, both poly-A and poly-T sequences are similarly depleted of nucleosomes in an approximately symmetric fashion, both in the presence and absence of a WCE. However, upon addition of ATP, which activates CRs present in the WCE, the sequence becomes further depleted, but in an asymmetric fashion; a nucleosome becomes well-positioned 5′ to the poly-A sequence, but not 3′, and the NFR is offset 5′ to the poly-A sequence, similar to the trend observed *in vivo* (**Figure 2**).

**Figure 3:**
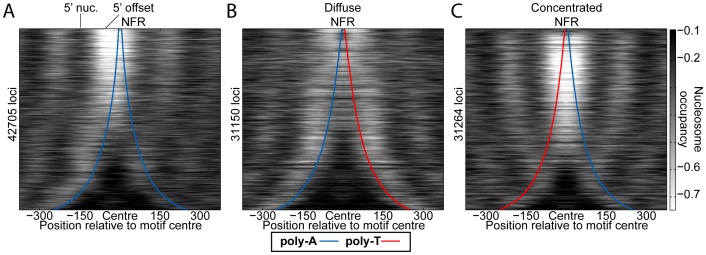
The different poly-A/poly-T arrangements result in vastly different nucleosome occupancy outcomes. *In vivo* nucleosome occupancy [7] (heatmap) surrounding all instances of (**A**) poly-A/poly-A, (**B**) poly-A/poly-T, and (**C**) poly-T/poly-A combinations in the yeast genome separated by no more than 500 bp. Red and blue curves represent the outer motif edges of poly-Ts and poly-As, respectively. Note that the poly-T/poly-T combination is a mirror image of the poly-A/poly-A data.

We next asked how nucleosomes were positioned around the three possible distinct arrangements of poly-A sequences (poly-A/poly-A, poly-A/poly-T, poly-T/poly-A). *In vivo* [7], when two poly-A elements are within ∼60 bp, a strong NFR that is offset 5′ to the poly-A sequences generally results (**Figure 3A**). The poly-A/poly-T arrangement is typically much less depleted between the two motifs and yields two NFRs; one 5′ to the poly-A and the other 3′ to the poly-T (**Figure 3B**). The poly-T/poly-A combination results in the most robust NFR (**Figure 3C**), which could explain why this arrangement is preferred in yeast promoters. Further, in all cases, nucleosomes tend to be more well-positioned 5′ to poly-A sequences (3′ to poly-T). *In vitro*, in the absence of WCE and ATP [1], there is little difference between the three possible poly-A/poly-T arrangements and, in general, nucleosomes are depleted symmetrically around each poly-dA:dT element (**Figure S2** in **File S1**). We note that the occupancy bias surrounding poly-dA:dT tracts in the presence of active chromatin remodelers is unlikely to result from differences in the nucleosome isolation/quantification procedures because the same procedures were used to generate all *in vitro* data [1], but the bias occurs only when WCE and ATP are both present (**Figure 2**). Further, the nucleosome occupancy bias surrounding poly-A/poly-T combinations is consistent between *in vivo* datasets that use different approaches for crosslinking (sulfhydryl [8], formaldehyde [1], [7]), cleavage (peroxide-mediate [8], MNase [1], [7]), and quantification (microarray [7], sequencing [1], [8]; see **Figure S3** in **File S1**).

**Figure 4:**
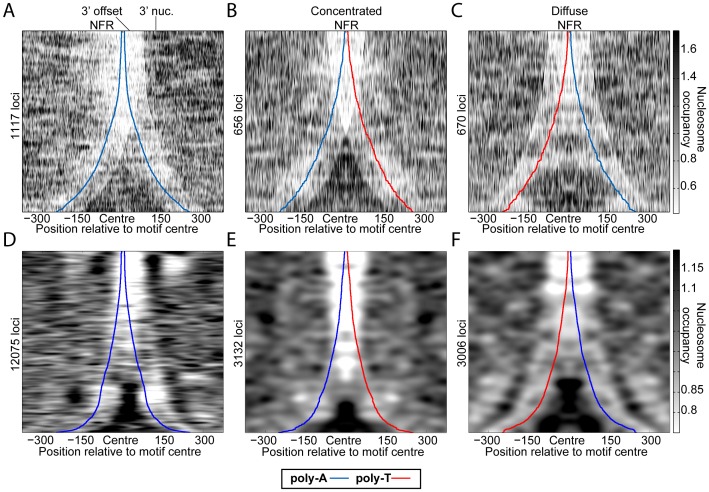
Mammalian nucleosome occupancy is also biased surrounding poly-As and poly-Ts, but the trend is opposite to yeast. *In vivo* nucleosome occupancy for (**A–C**) regions with available high-resolution nucleosome data from mouse Th1 cells [9] and (**D–F**) non-repetitive regions on chromosome 22, for human granulocytes [10] (heatmaps) surrounding all instances of (**A**, **D**) poly-A/poly-A, (**B**, **E**) poly-A/poly-T, and (**C**, **F**) poly-T/poly-A combinations. Gaussian smoothed between rows (SD = 10 and 50, for mouse and human, respectively). The distinct transitions from light to dark in the mouse data (**A-C**) result from using unsmoothed data, which corresponds roughly to nucleosome dyad occupancy (in this case the poly-A/poly-T bias was more obvious without smoothing). This distinct transition is presumably caused by the destabilization of nucleosomes as poly-dA:dT tracts are incorporated, and nucleosomes appear to be most unstable when the dyad is 69 bp from the proximal poly-dA:dT tract edge in human, mouse, and yeast (**Figure S4** in **File S1**).

We hypothesize that the CR-dependent asymmetric arrangement of nucleosomes surrounding poly-A elements reflects differences in the nucleosome translocation efficiency from upstream vs. downstream of poly-As. It is possible that such a difference could result from the different histone-DNA contacts of the two DNA-strands. However, mouse [9] and human [10], which have nucleosomes very similar to those of yeast (84% identical in histone fold domains, between mouse and yeast), display a trend opposite to yeast (**Figure 4**); poly-A/poly-T combinations tend to be more depleted than poly-T/poly-A combinations, two consecutive poly-As generally result in 3′-biased NFRs, and, overall, there appear to be a more robust nucleosome boundaries 3′ to poly-As (5' to poly-Ts). This observation suggests that specific factors (e.g. CRs) are responsible for differentiating between poly-As and poly-Ts. For example, poly-A tracts could prevent binding of CRs such that they can move a nucleosome towards poly-A sequences, but once there, the CR binds the DNA less efficiently and so cannot move it away. Indeed, previous studies have hinted that the DNA sequence could influence the repositioning of nucleosomes by CRs *in vitro*, but the mechanism, *in vivo* relevance, and sequence determinants of this phenomenon remained unknown [11], [12]. More detailed studies of nucleosome positioning in the presence or absence of different CRs will be needed to determine the specificities of these CRs.

Our data indicate that poly-A sequences form an asymmetric barrier to CR-mediated nucleosome transit, that this asymmetry is used in yeast promoter architecture, and that the same sequences are used differently in mammals. This phenomenon helps explain part of the discrepancy between *in vitro* and *in vivo* nucleosome occupancy and indicates that the DNA sequence may play a greater role in positioning nucleosomes in the cell than previously appreciated. More complex models of nucleosome occupancy that account for CR-mediated nucleosome transit may be needed to fully explain nucleosome occupancy in the dynamic environment of the cell.

## Methods

### Definition of poly-A/poly-T

For **Figure 1** and **Figure 2**, we defined a poly-A element as any instance of five As in a row in the genome, with poly-T defined similarly. For **Figure 1**, we calculated the expected occurrence of poly-As and poly-Ts by using the nucleotide frequency at every base pair in the region to calculate the proportion of promoters expected to contain a poly-A or poly-T sequence at any given position. For **Figure 2**, we only considered non-overlapping instances.

The poly-A/poly-T combinations in **Figure 3**, and **Figure S2** and **S3** in **File S1** were derived by identifying all maximal poly-A and poly-T elements of at least 5 bp in the yeast genome and considering only those motif pairs whose (outer) edges lie within 500 bp and that have no additional poly-dA:dT tracts between them. **Figure 4** was created similarly, but only considering BAC-enriched regions for mouse data (i.e. regions for which high-resolution occupancy data are available) and only non-repetitive (by repeatmasker) regions of chromosome 22 for human. For **Figure S4** in **File S1**, only poly-A tracts of exactly length 5 were considered. In all cases, we used the NCBI v37 mouse genome, hg18 human genome, and R64 yeast genome.

### Nucleosome occupancy normalization

For the data displayed in **Figure 2**, **Figure 4** (**D–E**), and **Figure S2** and **S3** in **File S1** (*in vitro* and *in vivo* yeast sequencing data [1], [8], and MNase-digested chromatin from human granulocytes [10]), we smoothed the data within each locus (Gaussian, SD = 20 bp), while for the data in **Figure 3** (yeast microarray data [7]) and **Figure 4** (**A–C**) (mouse Th1 sequencing data, representing the centres of 147 bp fragments isolated from native, MNase-digested chromatin [9]), we performed no such smoothing. Smoothing the data in this way makes it correspond more closely to nucleosome occupancy by distributing the dyad occupancy (nucleosome centre position) over the area covered by a nucleosome. We did not smooth the mouse data because doing so obscured the poly-A/poly-T bias. We noted that the sequencing data (**Figure 2**, **Figure 4**, and **Figure S2–S4** in **File S1**) displayed significant variation in the number of reads per locus, so, for these data, we scaled each locus so that they each had a comparable numbers of reads and threw out any loci containing fewer than 40 (yeast *in vitro*; **Figure S2** and **S4A** in **File S1**), 400 (mouse *in vivo*; **Figure 4A-C** and **Figure S4C** in **File S1**), or 100 reads (human *in vivo*, **Figure 4D–F**, **Figure S4B** in **File S1**). For **Figure 3**, **Figure 4**, and **Figure S2** and **S3** in **File S1,** we also smoothed between loci to emphasize the overall occupancy trend (Gaussian, SD = 50, except for **Figure 4** **A–C**, for which we used SD = 10).

### Significance of poly-As and poly-Ts in promoter regions

To gauge the significance of the distribution of poly-As and poly-Ts in promoter regions, we generated “random-sequence promoters” (in equal proportion to the number of actual promoters analyzed) where, at every base position, that base had the same probability of being an A or T as the actual frequency of that base at that position. We repeated this procedure 10^6^ times and each time counted the number of occurrences of 5 Ts or 5 As in a row within the −115:−75 and −75:−35 regions (relative to the TSS), respectively, but we found no randomly generated set of promoters with as extreme an occurrence of poly-As and poly-Ts in these regions as observed *in vivo* (max simulated = 1653 and 480, actual = 4919 and 2449 for A5 and T5, respectively).

### Significance of nucleosome bias surrounding poly-dA:dTs

To gauge the significance of the biased distribution of nucleosomes surrounding poly-As and poly-Ts, we compared the distribution of normalized (as described above) reads surrounding these sequences within each experimental condition. We used the two-tailed (Mann-Whitney) rank sum test to gauge the significance of the difference in occupancy for poly-As compared to poly-Ts at equivalent positions relative to the poly-A/T. The result is plotted in **Figure S1** in **File S1**, along with the Bonferroni multiple hypothesis correction significance threshold.

## Supporting Information

File S1 Supplementary figures and figure legends.
(DOC)
