# Supplementary material for: Poly-dA:dT Tracts Form an In Vivo Nucleosomal Turnstile
Source: PLoS One. 2014 Oct 29;9(10):e110479. doi: 10.1371/journal.pone.0110479 (PMC4212969; doi:10.1371/journal.pone.0110479)
Supplement: File S1 — Supplementary figures and figure legends. (DOC) [file pone.0110479.s001.doc]

# SUPPLEMENTARY FIGURES

**
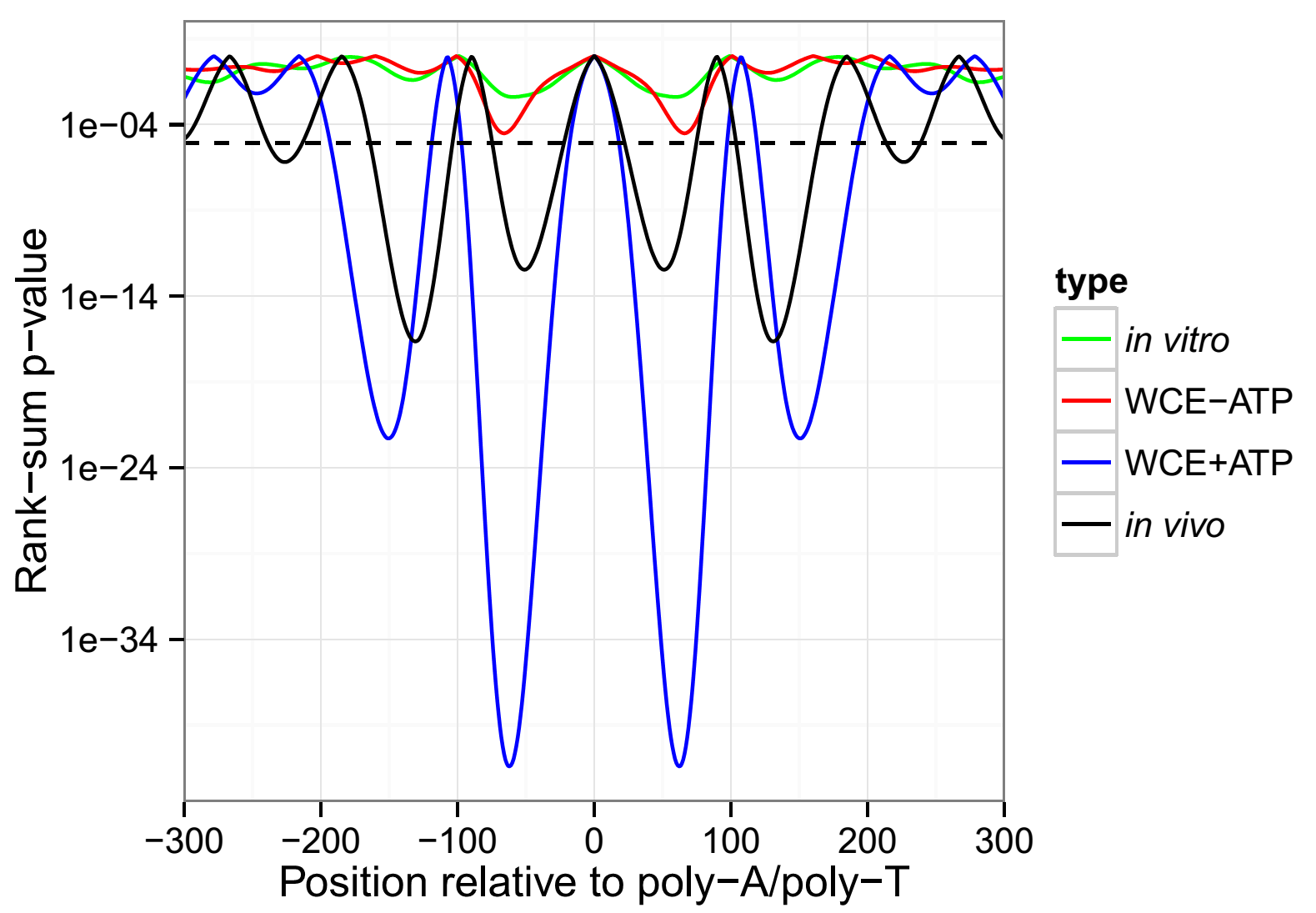
**

**Supplementary Figure 1**: Rank sum p-values comparing the nucleosome occupancy distributions relative to poly-As and poly-Ts for each base pair across the region displayed in **Figure 2**, for each experimental condition. The dashed line indicates the significance threshold after correcting for multiple hypothesis testing.


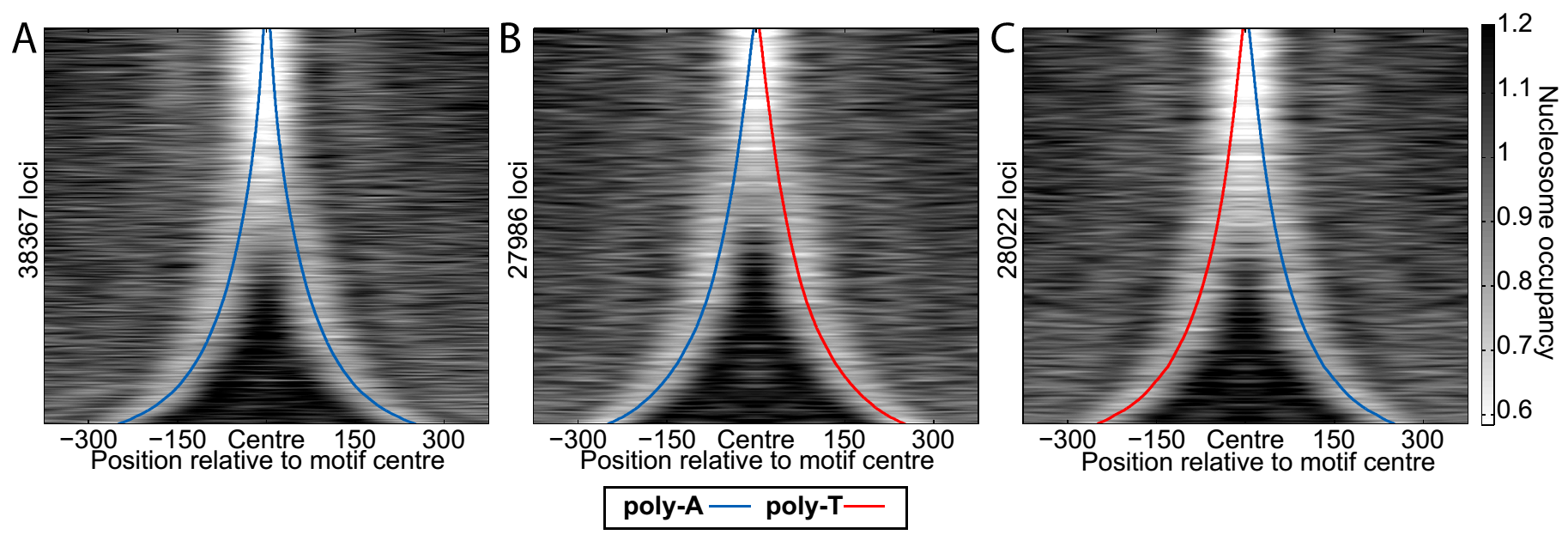


**Supplementary Figure 2**: *In vitro* nucleosome occupancy (Salt Gradient Dialysis) for the three possible arrangements of poly-A elements, for all instances in the yeast genome separated by no more than 500 bp. In each plot, the heatmaps represent normalized *in vitro* nucleosome occupancy , smoothed between rows (Gaussian, SD=50), sorted by the distance between the outer motif edges (shown in red and blue curves), for (**A**) poly-A/poly-A, (**B**) poly-A/poly-T, (**C**) poly-T/poly-A. Loci are identical to those shown in **Figure 3**, with the exception of the rows removed because they contained too few reads (see **Methods**).


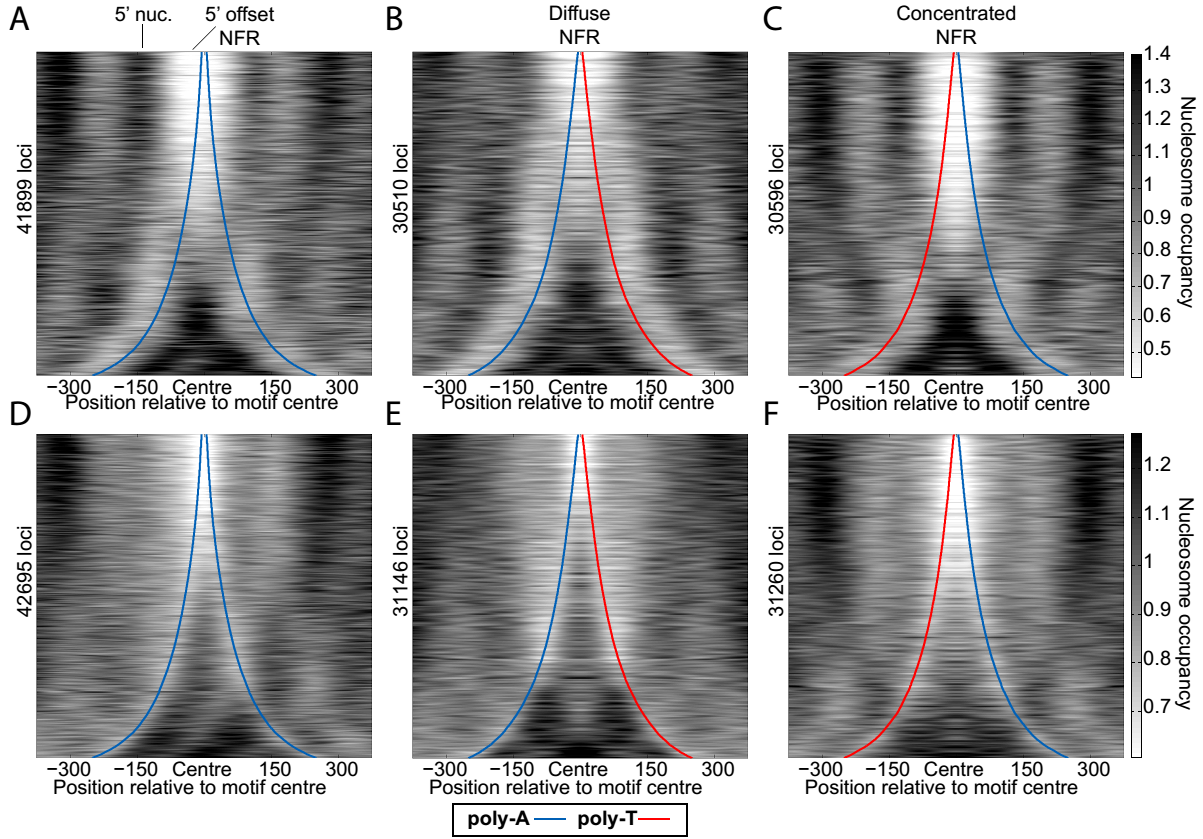


**Supplementary Figure 3:** Different nucleosome datasets also show a bias for the different poly-A/poly-T arrangements in yeast. *In vivo* nucleosome occupancy (heatmap) surrounding all instances of (**A,D**) poly-A/poly-A, (**B,E**) poly-A/poly-T, and (**C,F**) poly-T/poly-A combinations in the yeast genome separated by no more than 500 bp. Red and blue curves represent the outer motif edges of poly-Ts and poly-As, respectively. (**A-C**) represent data from and (**D-F**) are from .


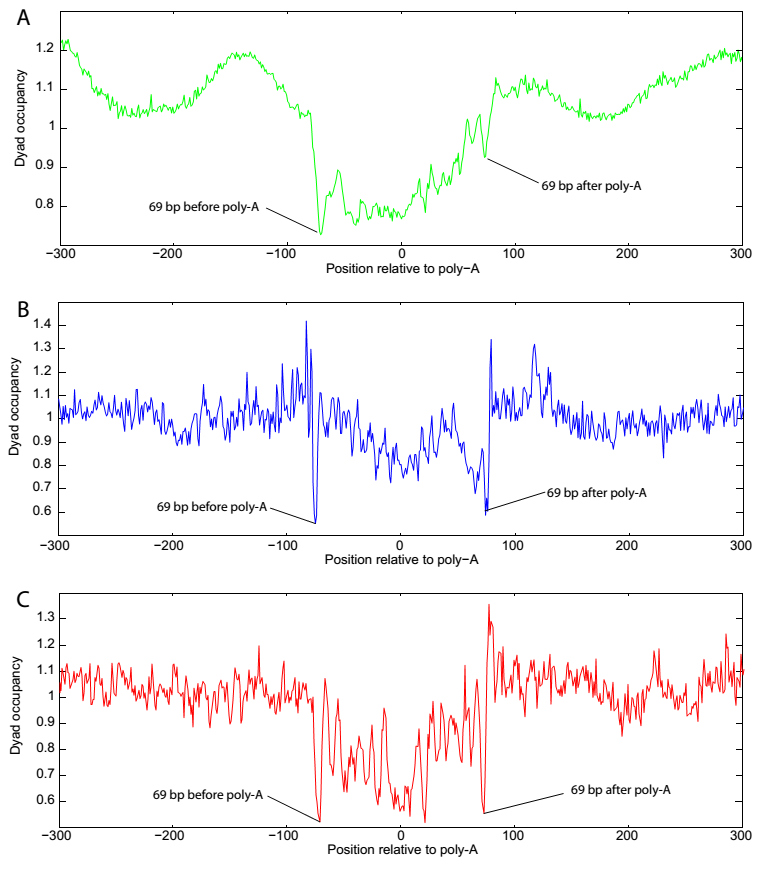


**Supplementary Figure 4**: Poly-dA:dT tracts form a nucleosome barrier in yeast, human, and mouse. The above plots show the average *in vivo* dyad occupancy across all poly-A(5) tracts (**A**) in yeast , (**B**) human chromosome 22 , and (**C**) BAC-enriched regions in mouse . In each species, there is a depletion in dyad signal where the poly-dA:dT tract is 69 bp away from the dyad.

# SUPPLEMENTARY REFERENCES

1. Zhang Z, Wippo CJ, Wal M, Ward E, Korber P, et al. (2011) A packing mechanism for nucleosome organization reconstituted across a eukaryotic genome. Science 332: 977-980.

2. Brogaard KR, Xi L, Wang JP, Widom J (2012) A chemical approach to mapping nucleosomes at base pair resolution in yeast. Methods Enzymol 513: 315-334.

3. Valouev A, Johnson SM, Boyd SD, Smith CL, Fire AZ, et al. (2011) Determinants of nucleosome organization in primary human cells. Nature 474: 516-520.

4. Yigit E, Zhang Q, Xi L, Grilley D, Widom J, et al. (2013) High-resolution nucleosome mapping of targeted regions using BAC-based enrichment. Nucleic Acids Res 41: e87.
